# Changes of bone turnover markers and serum PTH after night or morning administration of zoledronic acid in breast cancer patients with bone metastases

**DOI:** 10.1038/sj.bjc.6604390

**Published:** 2008-05-27

**Authors:** D Generali, A Dovio, M Tampellini, M Tucci, S Tedoldi, M Torta, S Bonardi, G Allevi, S Aguggini, M Milani, A L Harris, A Bottini, L Dogliotti, A Angeli, A Berruti

**Affiliations:** 1 Breast Unit, Azienda Ospedaliera Istituti Ospitalieri, Cremona, Italy; 2 Weatherall Molecular Oncology Laboratories, Institute of Molecular Medicine, John Radcliffe Hospital, University of Oxford, Oxford, UK; 3 Medicina Interna e, Dipartimento di Scienze Cliniche e Biologiche, Università degli Studi di Torino – Azienda Ospedaliera San Luigi di Orbassano, Orbassano, Italy; 4 Oncologia Medica, Dipartimento di Scienze Cliniche e Biologiche, Università degli Studi di Torino – Azienda Ospedaliera San Luigi di Orbassano, Orbassano, Italy

**Keywords:** breast cancer, bone metastasis, zoledronic acid, parathyroid hormone, bone turnover, circadian rhythm

## Abstract

Persistent circadian rhythm of bone turnover in bone metastatic breast cancer suggests greater skeletal retention of bisphosphonates if administered in the night. We assessed differential effects of night *vs* morning administration of zoledronic acid (ZA) on bone turnover. Forty-four breast cancer patients with bone metastases were randomised to receive intravenous ZA (4 mg) at 1100 or 2300 hours every 28 days for four times. Urinary concentration *N*-telopeptide of type-I collagen (NTX) and deoxypyridinolines, and serum C-telopeptide of type-I collagen (CTX), bone alkaline phosphatase (ALP), osteocalcin and Parathyroid hormone (PTH) was measured in the morning at baseline and after 4, 7, 14, 28, 56 and 84 days. Urinary ZA concentration was also measured. Zoledronic acid caused significant decreases of NTX and CTX (*P*<0.001), without any difference in percent changes between night and morning arms. Bone ALP and osteocalcin were also significantly affected by ZA (*P*=0.001), without any difference between arms. Parathyroid hormone significantly increased in both the arms; PTH increase was lower in the night arm (*P*=0.001). From the second administration onwards, urinary ZA level was significantly higher in the night arm (*P*<0.01). Administration of ZA at two opposite phases of the circadian cycle causes similar changes of bone-turnover marker levels, but has differential effects on the level of serum PTH.

Bone metastases are frequent in patients with advanced breast cancer. A complex crosstalk between cancer cells and cells of the bone microenvironment leads to disruption of the normal coupling between formation and resorption, eventually resulting in osteolytic, osteoblastic or mixed appearances. In the vast majority of cases, increased osteoclast activity predisposes patients to undergo adverse skeletal events such as fractures, hypercalcaemia and spinal cord compression (Kakonen and Mundy, 2003).

Bone-turnover markers provide valuable indirect information regarding the changes occurring in the basic multicellular units as a consequence of cancer colonisation and progression, and the effects of therapy. Breakdown products of type-I collagen, such as pyridinoline, deoxypyridinoline (DPD), N- and C-terminal telopeptides (NTX and CTX), are established markers of bone resorption (Pagani *et al*, 2005; Seibel, 2005). They are released into the circulation and are ultimately excreted in the urine. They are often elevated in patients with bone metastases irrespective of the radiological appearance of lesions; remarkably, they correlate with extent of metastases, bone pain (Berruti *et al*, 1999) and prognosis, including onset of adverse skeletal events (Brown *et al*, 2003, 2005; Coleman *et al*, 2005; Mundy, 2005). Bone resorption has a circadian rhythm, with levels of all relevant urinary and serum markers peaking in the early morning hours (Bjarnason *et al*, 2002). We have recently demonstrated that this rhythmicity is roughly maintained in patients with bone metastatic breast cancer (Generali *et al*, 2007).

Bisphosphonates are effective in preventing skeletal complications in patients with cancer bone disease (Pavlakis *et al*, 2005). Zoledronic acid (ZA), the most potent bisphosphonate available nowadays, is widely used for this purpose (Rosen, 2004; Rosen *et al*, 2004). Pertinently, the clear-cut fall of urinary NTX level after ZA administration has been associated with reduction of adverse skeletal events (Coleman *et al*, 2005). Since bisphosphonates bind preferentially to the exposed hydroxyapatite at the resorption sites (Lin, 1996), a higher percentage of the administered dose may be taken up by the bone when the resorptive activity is higher, that is, during night. In a recent investigation, Cremers *et al.* (2005) have focused on the significant relationship between skeletal retention of pamidronate and the antiresorptive effect of the drug. To address whether nocturnal administration of ZA could lead to differential effects with respect to conventional morning administration, in this study we have measured the level of bone-turnover markers, serum calcium and parathyroid hormone (PTH) in bone metastatic breast cancer patients at first relapse randomised to receive the same regimen of ZA at 1100 or at 2300 hours, that is, at two opposite phases of the circadian cycle.

## MATERIALS AND METHODS

### Study population

Forty-four consecutive patients (median age 62, range 32–77 years) with bone metastatic breast cancer, consecutively recruited at a single institution (Breast Unit, Cremona, Italy), were enrolled into the study between January and December 2002. Patients met the following inclusion criteria: histologically diagnosed breast adenocarcinoma at first disease relapse; at least one apparent metastatic site in bone (confirmed by conventional radiography, computed tomography and/or magnetic resonance imaging) and performance status between 0 and 2. Exclusion criteria were as follows: chemotherapy or endocrine therapy for advanced disease started before study entry (previous chemotherapy or endocrine therapy, administered in an adjuvant setting, should have been interrupted for at least one year); previous radiation therapy on bone lesions; previous bisphosphonate treatment; concomitant diseases known to affect bone; liver metastases; biochemical evidence of renal or hepatic dysfunction. All patients were post-menopausal, the menopause having been physiological in 31 and iatrogenic in 13 of them.

The study was performed in accordance with current guidelines on good clinical practice in clinical research and the Declaration of Helsinki and subsequent integrations. All subjects provided written, informed consent, and the study was approved by the local ethical committee.

### Protocol

Patients were randomised to receive ZA (4 mg diluted in 100 ml saline, intravenously, in about 15 min) either in the morning (approximately at 1100 hours) or in the night (approximately at 2300 hours), every 4 weeks for 4 months.

Serum levels of calcium, PTH, bone alkaline phosphatase (ALP), osteocalcin, CTX, and urinary NTX and DPD, were measured, respectively, in a blood sample drawn in the morning at about 0800 hours and in a second-void urine sample, at baseline (before starting ZA), and thereafter at days 4, 7, 14, 28, 56 and 84. All patients also had serum 25-hydroxyvitamin D level assessed at baseline. Zoledronic acid excretion was determined in the second-void urine samples collected at days 4, 7, 14, 28, 56 and 84. From the second ZA administration onwards, all patients received a combination regimen of intravenous vinorelbine (30 mg m^−2^ on days 1 and 8, every 21) plus oral capecitabine (2000 mg m^−2^ on days 1 to 14 every 21). During the study period no patient received daily calcium and vitamin D supplementation.

At baseline, disease extent in bone was scored by arbitrarily dividing the skeleton into the following regions: skull, cervical, dorsal, lumbar spine-sacrum, right leg, left leg, right arm, left arm, right ribs, left ribs, sternum, right scapula and clavicula, left scapula and clavicula, right pelvis and left pelvis. The sum of data from the involved areas was considered; in order to limit the distribution of this variable, patients with more than 10 segments were scored as 10.

Bone pain was evaluated at baseline and before each ZA administration (i.e., after 28, 56 and 84 days) using a validated pain questionnaire, as previously reported (Berruti *et al*, 2000). The questionnaire items included performance status, analgesic consumption and mobility, with a resulting pain score of 0–19.

### Biochemistry

A sampling cannula was inserted into a vein in the antecubital fossa. At each time point the initial 2.5 ml were discarded and a 5-ml blood sample was obtained. Routine biochemical analyses were performed with fresh samples; other aliquots were stored at −20°C before being analysed (within 6 months).

Serum calcium, creatinine, albumin and total ALP, and urinary calcium and creatinine levels were measured by standard automated analytical procedures (Modular P/P; Roche). Serum calcium levels were always corrected for serum albumin. Serum PTH (intact molecule) level was measured by an immunoradiometric assay (IRMA; Scantibodies Laboratory, Santee, CA, USA). Bone ALP level was measured by precipitation with wheat germ lectin (Iso-ALP; Roche Diagnostics GmdH, Mannheim, Germany). Osteocalcin concentration was measured by radioimmunoassay (RIA) using a monoclonal antibody that binds intact osteocalcin and fragments 1–43, 20–49 and 20–44 (Osteocalcina Myria; Technogenetics, Milan, Italy). 25-Hydroxyvitamin D was measured by RIA (Immunodiagnostic Systems, Boldon, UK). Deoxypyridinoline level was measured by high-performance liquid chromatography (Bio-Rad, Milan, Italy), while CTX and NTX levels were measured by ELISAs (Nordic Bioscience Diagnostic, Herlev, Denmark; and Osteomark, Ostex International, Seattle, WA, USA, respectively). Zoledronic acid level in urine was measured at a Novartis Pharma laboratory (Rueil Malmaison, France), according to a previously described highly sensitive RIA (Legay *et al*, 2002). Urinary marker levels were calculated relative to levels of urinary creatinine.

Measuring ranges, minimum detectable concentrations, intra- and inter-assay coefficients of variation were as follows: osteocalcin: 2.2–64.3 ng ml^−1^, 0.3 ng ml^−1^, 3.5 and 5.6%; bone ALP: 5–2000 U l^−1^, 5 U l^−1^, 0.5 and 2.2%; CTX: 0.156–2.562 ng ml^−1^, 0.01 ng ml^−1^, 2.5 and 9%; NTX: 30–3000 nM, 20 nM, 4.4 and 6.9%; DPD: 7–300 nM, 6 nM, 11 and 14%; PTH: 15–2000 pg ml^−1^, 1.8 pg ml^−1^, 3.2 and 5.8%; ZA: 1–1000 ng ml^−1^, 5 ng ml^−1^, 16–21 and 6.7–8%. All assays were performed in duplicate. Samples from a single subject were assayed within a single analytical session.

### Statistical analysis

Comparisons of qualitative and continuous variables were by *χ*^2^ and Mann–Whitney *U*-tests, respectively. With regard to bone-turnover markers, due to inter-individual differences in the baseline levels, changes were assessed at each time point as both absolute and percent values. With regard to PTH, it is held that levels in the peripheral blood undergo rapid oscillations due to ultradian rhythmicity, characterised by numerous secretory bursts per hour. Therefore, percent changes could be quite different as a function of a single baseline measurement occurring at the peak or the bottom of a burst lasting only few minutes. We decided to use absolute changes for this variable and to analyse also the area under the curve (AUC) calculated by trapezoidal method (Allison *et al*, 1995).

The time profiles were analysed by a multivariate analysis of variance for repeated measurements according to a mixed random effect model, which uses the covariance structure also for estimating the model parameters (SAS PROC MIXED with REPEATED statement and no RANDOM statement). Covariance matrix components were separately estimated for the two treatment arms. All *P*-values reported were two-sided; a 0.05 limit was chosen for statistical significance. Only subjects with all values were included in the model fitting. Time distances were considered as equally spaced in the model.

Since bone resorption markers are significantly correlated with disease extent in bone (Berruti *et al*, 1999), relevant data were also analysed after adjusting for the number of bone segments involved. All statistical analyses were performed using SAS software version 8.2.

## RESULTS

Twenty-two patients were randomised to each treatment arm. Patients’ characteristics are outlined in Table 1.

Zoledronic acid administration was well tolerated in both treatment arms. Twenty patients (45.4%), 10 for each treatment arm, experienced acute-phase reactions lasting no more than a few days after ZA administration. Mean serum creatinine level did not vary at any time point in either treatment arms. Zoledronic acid administration led to a decrease in bone pain without any difference between arms.

### Effect of morning *vs* night ZA administration on the markers of bone turnover

**Figure 1:**
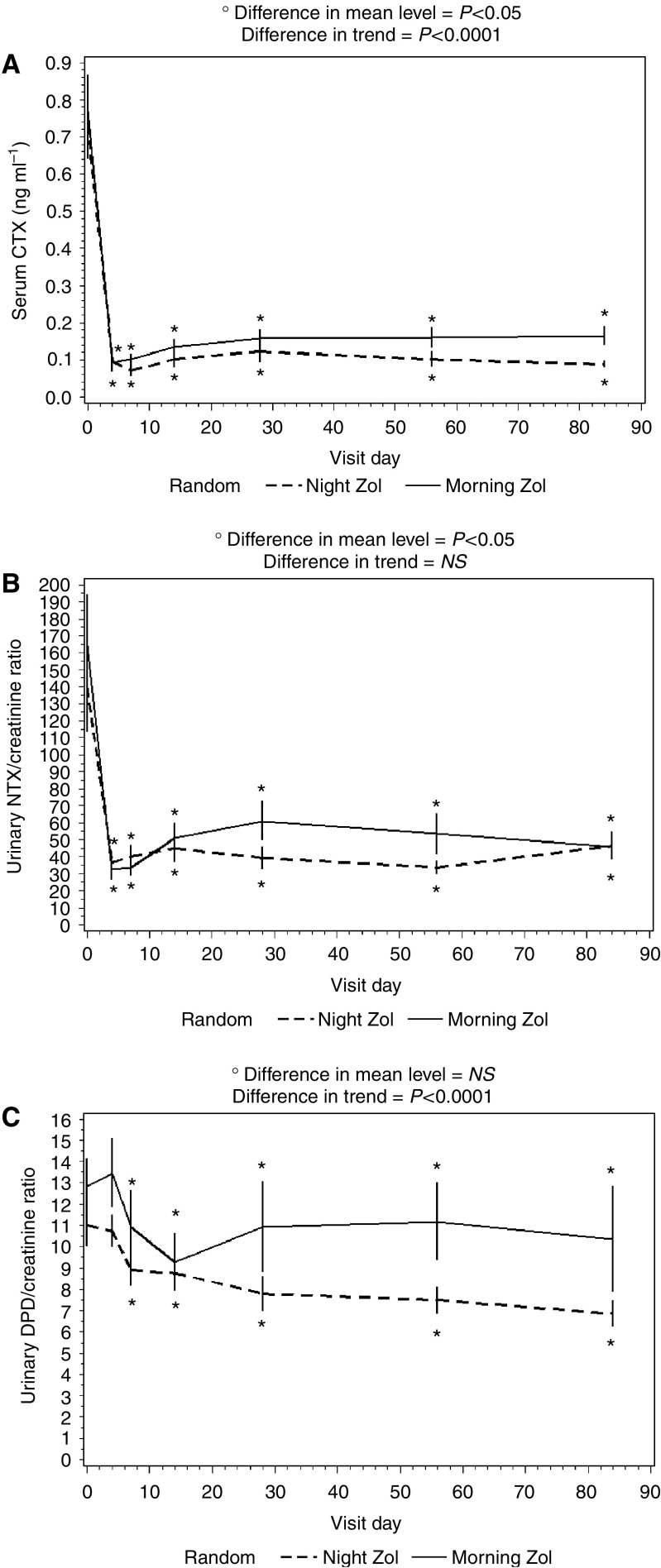
Serum CTX levels (**A**) and urinary NTX/creatinine (**B**) and DPD/creatinine (**C**) ratios after ZA administration. Data are presented as mean±s.e.; ^*^P<0.05 *vs* baseline.

As shown in Table 2, no difference in the levels of bone resorption and formation markers was observed between the treatment arms at baseline. When both arms were considered together, ZA administration led to significant decreases of urinary NTX (*P*<0.001) and serum CTX (*P*<0.0001) levels, but not of urinary DPD. The ANOVA performed on the levels of bone resorption markers obtained at each time point yielded the following results (Figure 1). Differences in mean absolute levels between the treatment arms were significant for serum CTX (*P*=0.05) and urinary NTX (*P*=0.05), but failed to attain the statistical significance when data were expressed as percent variations. The profiles of mean serum levels of CTX and urinary excretion of DPD of the two treatment arms showed progressively divergent trends over time (tests for interaction *P*=0.0001 and *P*=0.0001, respectively), whereas the profiles of urinary NTX did not (Figure 1). Statistical analysis yielded the same results after adjusting for disease extent in bone.

**Figure 2:**
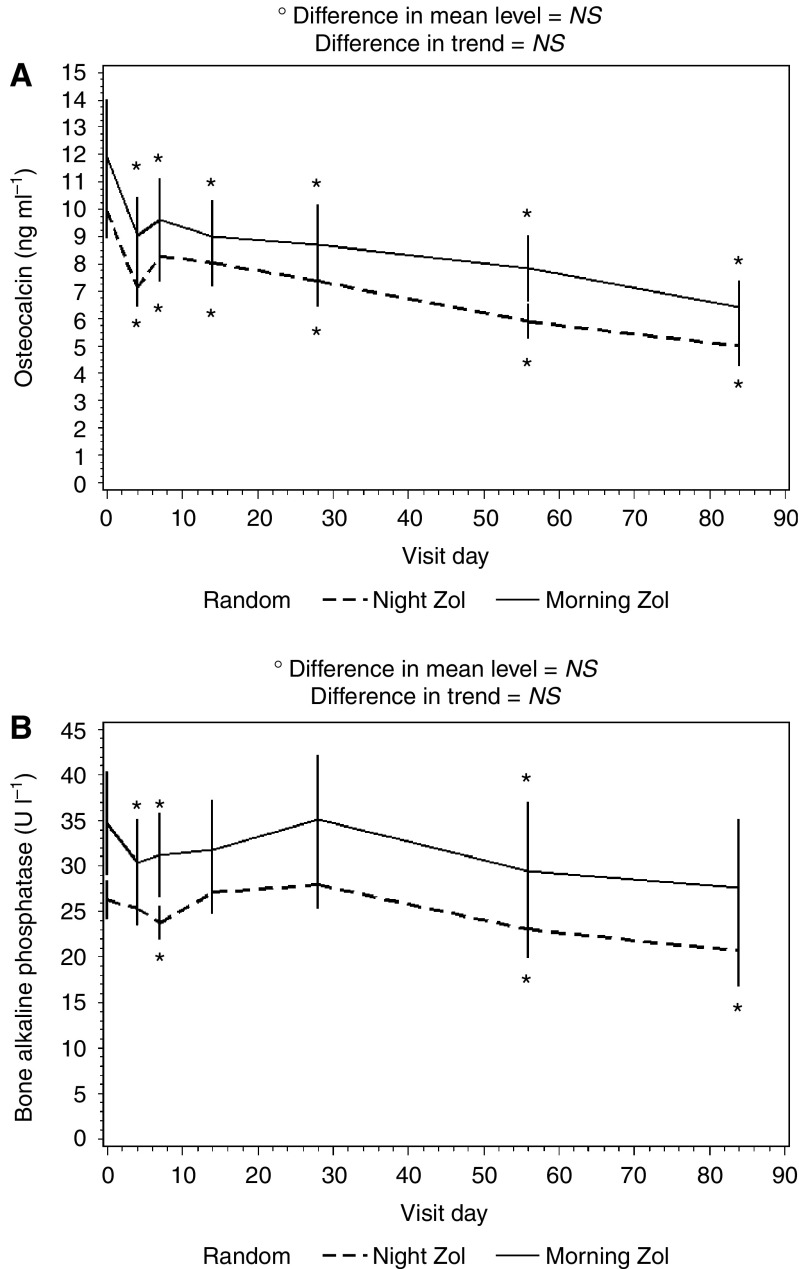
Serum osteocalcin (**A**) and bone ALP (**B**) levels after ZA administration. Data are presented as mean±s.e.; ^*^P<0.05 *vs* baseline.

As far as bone formation markers were concerned, when both arms were considered together, both bone ALP and osteocalcin significantly (*P*=0.001 for both variables) changed after ZA administration. Osteocalcin showed a substantial decrease (Figure 2A), whereas bone ALP showed an initial decrease followed by transient recovery to baseline and subsequent sustained decrease (Figure 2B). The time profiles of both markers were analogous in both treatment arms.

### Effect of morning *vs* night ZA administration on serum calcium and PTH level

**Figure 3:**
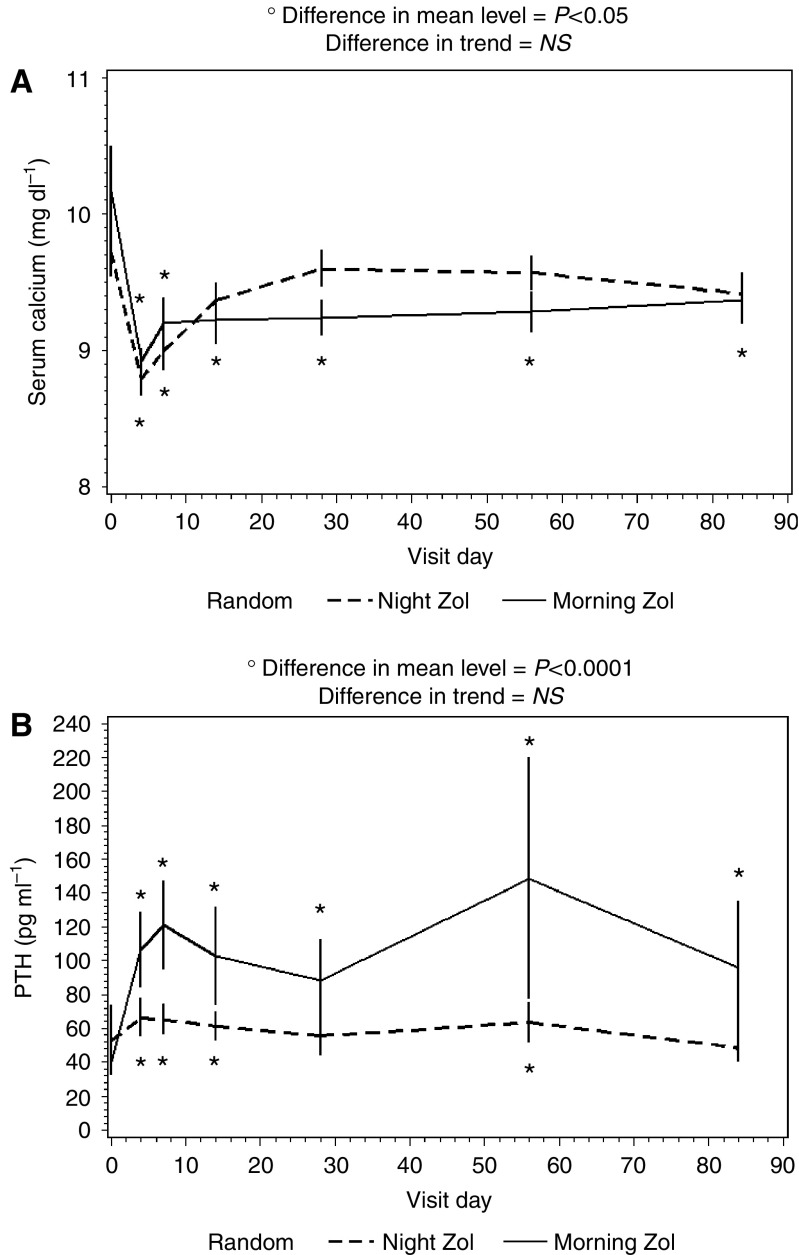
Serum PTH (**A**) and calcium (**B**) levels after ZA administration. Data are presented as mean±s.e.; ^*^P<0.05 *vs* baseline.

At baseline, patients randomised in the two treatments arms did not differ in terms of serum calcium, PTH and 25-hydroxyvitamin D concentrations (Table 2). When the two arms were considered together, corrected serum calcium decreased and serum PTH increased after ZA administration (*P*<0.001). When looking at differences between the two profiles obtained by giving ZA at the opposite phases of the 24-h cycle, serum calcium levels were similar in both the arms in the early period, whereas differing at days 28 (*P*<0.05) and 56 (*P*=0.05), and again overlapping at day 84 (Figure 3A); however, absolute decremental AUCs were similar in the two arms (*P*=0.19). The time profiles of mean PTH values showed a similar trend in both arms, yet the nocturnal regimen led to a significant lower PTH increase as assessed both by ANOVA (*P*=0.001; Figure 3B) and absolute incremental AUC (*P*=0.08). No significant correlation was found between calcium and PTH AUCs.

### Effect of morning *vs* night ZA administration on urinary ZA

Whereas similar patterns were noticed in both arms during the first month, from the second administration onwards mean drug levels before subsequent infusion were significantly higher in patients randomised to receive ZA in the night with respect to those receiving the drug in the morning (*P*<0.01).

## DISCUSSION

Current knowledge from experimental studies supports the notion that there is a vicious circle at the bone metastatic site where cancer cells stimulate osteoclast-mediated bone resorption, whereas bone-derived factors released from the resorbed bone promote metastasis growth (Kakonen and Mundy, 2003). A clinical consequence of this notion is the well-recognised use of bisphosphonates as an important feature of the therapy of patients with bone metastases. A direct association between the extent of skeletal retention of intravenously administered pamidronate and suppression of bone resorption rate has been demonstrated recently in a series of breast cancer patients with bone metastases (Cremers *et al*, 2005). Baseline bone turnover is a major determinant of bisphosphonates skeletal uptake (Lin, 1996). In the present study, we have explored whether administration of ZA in the night, when osteoclast activity is rising, could result in a greater suppression of bone turnover as compared with suppression by the conventional morning administration.

We did not find differential patterns of either bone resorption or bone formation markers after ZA administration at opposite phases of the circadian cycle. Yet analysis of the decremental curves for serum CTX and urinary DPD yielded a significant trend towards greater antiresorptive effect in the group of patients receiving the drug at night. Even if this finding was not substantiated by the significance in percent decreases from baseline levels, it could be viewed in keeping with the concept that ZA is more effective at the time of greater osteoclastic activity. The administered dose is worth of attention. The widely used 4-mg/28-day schedule could be maximal at each time of the day. In a setting of a lower loading, such as that indicated in patients with impaired renal function, circadian susceptibility could better emerge and be of clinical relevance. However, we did not measure excretion of ZA in the very first days and perhaps, most importantly, we did not assess the whole-body retention of the drug (Cremers *et al*, 2005), which could have been informative in this regard.

Anyway, the most interesting finding of our study could be viewed in the differential response of PTH secretion as a function of circadian phase. The mechanisms accounting for our data are not obvious. Intravenous administration of potent bisphosphonates usually leads to hypocalcaemia and secondary hyperparathyroidism (which are mild and transient in many cases, but may persist for a long time in others), with serum PTH level increase depending on the status of vitamin D (Rosen and Brown, 2003; Breen and Shane, 2004; Caspar *et al*, 2004; Tanvetyanon and Stiff, 2006). The two arms did not differ in 25-hydroxyvitamin D levels. To the best of our knowledge, no data are available on the possible direct effect of bisphosphonates on the steep sigmoidal relationship between PTH levels and changes in the extracellular concentration of calcium (Brown, 1983). Whatever the mechanisms, this finding could be of clinical relevance. In a large population recruited in the registration trials of ZA, hyperparathyroidism after ZA administration has been recently associated with worse prognosis (Berruti *et al*, 2006). Moreover, in a retrospective analysis, PTH levels before treatment with ZA were higher in patients who subsequently developed osteonecrosis of the jaw with respect to those who did not; patients developing this severe complication also showed lower calcium and higher PTH levels throughout treatment with ZA (Ardine *et al*, 2006). It is unknown whether sustained excess PTH secretion in bone metastatic patients reflects poor clinical conditions independently of therapy, or whether it has a role in the disease progression due to direct or indirect effects on tumour growth.

To conclude, the smaller increase in PTH level after ZA administration in the night could be viewed as an additional piece of evidence supporting differential effects of the drug as a function of the administration hour. The clinical implications in terms of anti-resorptive efficacy and PTH level changes deserve to be further tested in specifically tailored protocols.

## Figures and Tables

**Table 1:** Clinical characteristics of patients

	**Morning arm**	**Night arm**
Number of patients	22	22
Age (years)	62 (33–77)	61 (39–74)
Bone disease extent	3 (1–10)	4 (1–10)
Pain score	2 (0–4)	1.5 (0–4)
		
*Performance status*		
0	16 (72.7%)	18 (81.8%)
1	3 (13.6%)	1 (4.5%)
2	3 (13.6%)	3 (13.6%)
		
*Metastatic disease sites*		
Bone only	13 (59.1%)	14 (63.6%)
Bone plus lung	5 (22.7%)	6 (27.3%)
Bone plus soft tissues	4 (18.2%)	2 (9.1%)
		
*Previous adjuvant treatments*		
Chemotherapy only	5 (22.7%)	6 (27.3%)
Chemotherapy+endocrine therapy	17 (77.3%)	16 (72.7%)

Data are means (95% confidence intervals) or numbers of patients (%). No statistical difference was found between morning and night arms by *χ*^2^ or Mann–Whitney *U*-test.

**Table 2:** Bone turnover marker, serum calcium, 25-hydroxyvitamin D and PTH level at baseline in the two treatment arms

	**Morning arm**	**Night arm**
Serum CTX (ng ml^−1^)	0.77 (0.56–0.97)	0.73 (0.54–0.91)
Urinary NTX/creatinine ratio	164.4 (102.2–226.6)	138.7 (85.6–191.8)
Urinary DPD/creatinine ratio	12.8 (10.1–15.6)	11.0 (8.9–13.1)
Serum bone ALP (U l^−1^)	34.6 (22.7–46.5)	26.3 (21.8–30.8)
Serum osteocalcin (ng ml^−1^)	11.9 (7.5–16.3)	9.9 (7.8–11.9)
Serum calcium (mg per 100 ml)	10.1 (9.5–10.7)	9.7 (9.4–10.1)
Serum PTH (pg ml^−1^)	43.1 (25.6–60.7)	50.7 (9.5–91.7)
Serum 25-hydroxyvitamin D (nM)	71.1 (54.9–87.3)	72.4 (56.7–88.0)

ALP=alkaline phosphatase; CTX=C-telopeptide of type-I collagen; DPD=deoxypyridinoline; NTX=N-telopeptide of type-I collagen; PTH=parathyroid hormone.

Data are means (95% confidence interval).
